# Inferring router ownership based on the classification of intra- and inter-domain links

**DOI:** 10.1038/s41598-023-32202-6

**Published:** 2023-03-29

**Authors:** Yan Liu, Yi Zhao, Xiaoyu Guo, Lian Liu

**Affiliations:** 1 Key Laboratory of Cyberspace Situation Awareness in Henan Province, Zhengzhou, China; 2 Investigation Technology Center PLCMC, Beijing, China

**Keywords:** Computer science, Information technology

## Abstract

Research on router ownership inference is central to many Internet studies, such as network failure diagnosis, network boundary identification, network resilience assessment, and inter-domain congestion detection. The existing router ownership inference method *bdrmapIT* has relatively few constraints on routers at the end of traceroute paths, resulting in some inference errors. In this paper, a router ownership inference method based on the classification of intra- and inter-domain links is proposed. In this method, the differentiating Internet Protocol (IP) address vector distance feature, the autonomous system relationship feature of the IP link, and the fan-in and fan-out features are designed to support the discrimination of IP link types. The use of additional information derived from the link type enriches the basis for router ownership inference and improves the accuracy of the inference result. Experimental results show that the accuracy reaches 96.4% and 94.6% on the two verification sets, respectively, which is 3.2–11.2% better than the existing typical methods.

## Introduction

One of the main terminologies for analyzing the Internet ecosystem is the Autonomous System (AS)^1^. An AS is a set of routers run by one or more network operators with a single and clearly defined routing policy^2^. Recently, the total number of ASes worldwide is 109,4371. Router ownership inference, the technology aiming to assign routers to their operating ASes, is a challenge in Internet topology research.

Accurate router ownership inference plays an essential role in many problem domains. First and foremost, it allows researchers to combine the router-level and AS-level Internet topologies, grasp the real connection details within and between ASes^3,4^, and then understand the composition of the Internet at a fine-grained level^5,6^. Besides, it helps network managers to maintain network performance, such as detecting congestion points that appear on links between ASes, analyzing the regularity of the Internet interdomain routing^7^ and inferring the delay on border links^8^. Additionally, it benefits network security researchers to identify AS border routers which are critical network infrastructures with great significance in application and safety.

In the actual Internet, multiple factors interfere with the accurate inference of router ownership. First, routers may respond to traceroute probes with different interface addresses, including third-party addresses^9^, which will interfere with router ownership inference. Second, the Internet Protocol (IP) address subnet space of the inter-AS connection usually comes from only one of the two ASes^10^, i.e., IP addresses sharing between ASes, resulting in it challenging to identify the inter-domain connection accurately. Additionally, considering security and competition factors, Internet operators tend to hide their network topologies, making it difficult to obtain some ground truth for modeling.

The current typical algorithm bdrmapIT^11^ achieved 91.8%-98.8% accuracy when mapping AS boundaries in two Internet-wide traceroute datasets. However, this method has relatively few available constraints when inferring routers at the end of traceroute paths, so there is still room for improvement in its accuracy. In response to this problem, in this study, we call routers that appear in the back part of traceroute paths and maybe in the same AS with traceroute destination ***last-AS*** routers. For ***last-AS*** routers, a Link Classification-based method for Router Ownership Inference (LCROI) is proposed. LCROI first classifies intra-domain and inter-domain links by establishing a probability model with the type of IP link as the hidden variable. After that, the information implied in the link type can increase available constraints when inferring ***last-AS*** routers resulting in better accuracy.

The main contributions of our work can be summarized as follows:

1. We introduced three distinctive features to differentiate between intra-domain and inter-domain links. Using these features, we developed a probability model with link type as the hidden variable to classify IP links.
2. A method for inferring router ownership, known as Link Classification-based Router Ownership Inference (LCROI), has been proposed. LCROI used link classification results to impose constraints on routers, resulting in higher accuracy in router ownership inference, as demonstrated in actual test results.

The rest of this article is organized as follows: the “Related work” section presents the related work on router ownership inference. In the “Analysis of bdrmapIT and problem definition” section, bdrmapIT is briefly analyzed and the problem of router ownership inference is defined. The “Proposed LCROI method” section describes the basic principles and main steps of the proposed method. The experiment and analysis are detailed in the “Experiments and results” section. Finally, the “Conclusion and Future Work” section concludes this article.

## Related work

Existing methods of router ownership inference rely on traceroute topology measurement, alias resolution, and IP-to-AS mapping technology. Routers have multiple interfaces with different IP addresses. Alias resolution technology can identify IP interfaces belonging to the same router. IP-to-AS mapping is a technology that maps IP addresses to their AS Numbers (ASN). The widely used IP-to-AS mapping method is to look up the origin IP-to-AS mapping table extracted from the Border Gateway Protocol (BGP) routing table, where each IP address is mapped to the origin AS of its longest matching prefix. A Multiple Origin AS (MOAS) conflict occurs when a particular prefix appears to originate from more than one AS.

According to the application scope of the method, existing router ownership inference methods can be divided into two categories: methods for a single AS and methods for the entire Internet, as shown in Table 1.

**Table 1:** Related works of router ownership inference.

Categories	Authors	Methods	Drawbacks
For a single AS	Luckie et al.^12^	Bdrmap: traceroute from VPs and topological analysis	Only available for the network hosting VPs and can be observed by VPs
For the entire Internet	Tangmunarunkit et al.^13,14^	Choose the most frequently assigned AS in the origin AS set	Simple and not accurate enough
	Chang et al.^3^	Intersection rule, majority rule, and hole-filling heuristic	Did not verify with actual information
	Claffy et al.^15^	Providers provide address spaces between themselves and their customers	Does not apply in all cases
	Huffaker et al.^16^	Five different heuristics: Single, Election, Neighbor, Customer, and Degree	The accuracy needs to be improved
	Pansiot et al.^17^	Router-to-AS algorithm based on probabilistic and empirical IP allocation rules	The scope of application of the method is limited
	Motamedi et al.^18^	IXP-assigned IPs and Valley-free heuristics, and some improved previous heuristics	There are cases where the owner AS cannot be inferred for the router
	Marder et al.^11^	BdrmapIT: combine bdrmap^12^ and MAP-IT^33^	Constraints available in annotating last hops are not enough

The router ownership inference method for a single AS first selects the target AS to deploy Vantage Points (VPs) for the traceroute probe. Then it infers the owner of routers inside the target AS and border routers inside neighboring ASes. Bdrmap proposed by Luckie et al.^12^ is for the single target AS. It first executes targeted traceroutes from VPs inside an AS and then combines the AS relationship data, using traceroute features and topology constraints to perform inference. It can only infer inter-domain connections related to the network hosting VPs and can be observed by VPs.

The router ownership inference method for the entire Internet uses heuristic methods to infer router ownership. Tangmunarunkit et al.^13,14^ chose the most frequently assigned AS in the origin AS set of the router. The method is simple and has insufficient accuracy. Chang et al.^3^ argued that a router is a border router if its interface addresses are assigned from multiple administration domains, and proposed the intersection rule, majority rule, and hole-filling heuristic. Their approach complements the method proposed by Tangmunarunkit et al.^14^. However, they did not verify their heuristics with any actual information of AS border routers. Claffy et al.^15^ believed that in provider-customer connections, the provider side provides the address space. This experience was used in the methods proposed by Huffaker et al.^16^, Pansiot et al.^17^, Motamedi et al.^18^, and others. Also, since it does not apply to all cases, other heuristics were supplemented to improve the inference accuracy. Huffaker et al.^16^ created an AS frequency matrix for each router. The matrix counted the number of interfaces (known and inferred) from each AS that appears on the router. Next, they described five different heuristics to produce an AS-router dual graph, including Single, Election, Neighbor, Customer, and Degree. The accuracy of their results needs to be improved. Pansiot et al.^17^ proposed a Router-to-AS algorithm based on probabilistic and empirical IP allocation rules to extract intra-domain topology from a large dataset collected with mrinfo. However, since only routers supporting Internet Protocol Version 4 (IPv4) multicast can reply to mrinfo, the scope of application of the method is limited. Motamedi et al.^18^ proposed Internet eXchange Point-assigned (IXP-assigned) IPs and Valley-free heuristics, and improved some of the previous heuristics as needed. If all of their heuristics fail to identify the AS owner, they would honor the original BGP-based AS ownership assignment for the concerned interfaces. Marder et al.^11^ created bdrmapIT, which combined bdrmap and their previous algorithm MAP-IT^33^ used for iteratively inferring inter-AS links at the interface-level graph, to infer router owners at the Internet scale. However, the inference accuracy can be improved somewhat for some routers.

## Analysis of bdrmapIT and problem definition

This section briefly introduces and analyzes the principle of the bdrmapIT^11^ method and gives a formal definition of the research problem in this paper.

### Analysis of bdrmapIT

**Figure 1:**
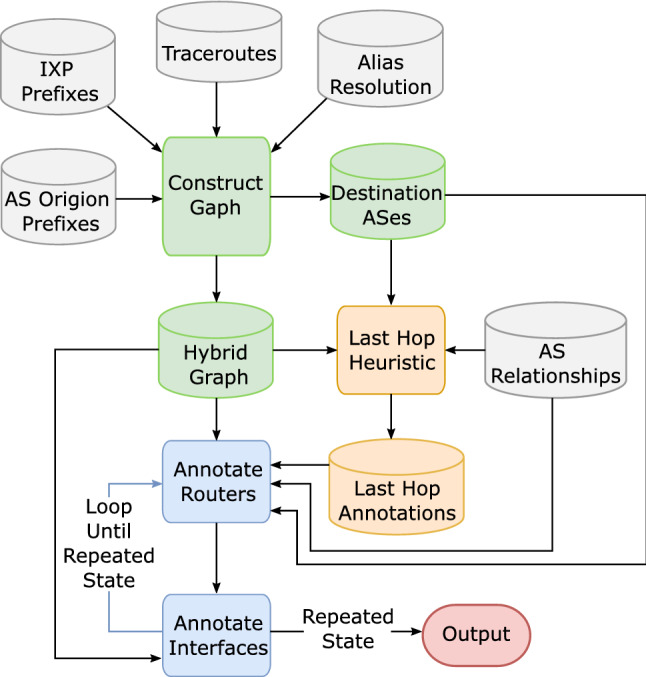
The flow chart of bdrmapIT^11^.

The bdrmapIT^11^ is a classical approach that implements router ownership inference at the Internet scale. When it maps AS boundaries on two traceroute data sets, the accuracy is more than 90%. bdrmapIT^11^ method mainly includes three phases: constructing the graph, annotating the last hops, and graph refinement. A router-level directed topology is established in the graph construction phase based on the IP-level topology from traceroutes after alias resolution. The second phase infers the operators of routers that appear only at the end of traceroutes. This phase provides topological context for mappings in the graph refinement phase. The graph refinement phase maps routers observed in the middle of at least one traceroute path to their ASes, and continuous corrections are made through iteration. Finally, accurate AS inference results of all routers are obtained. The primary process of bdrmapIT is shown in Fig. 1.

The bdrmapIT^11^ combines bdrmap^12^ and MAP-IT^33^ to adapt bdrmap which targets a network to the iterative improvement framework of MAP-IT. This reduces bdrmap’s requirements for the number and location of VPs, and expands its scope of application (from a specific network to any number of networks). Moreover, it optimizes the limitation that MAP-IT does not use alias resolution and only performs AS-level boundary recognition at the interface level. Although bdrmapIT has achieved excellent results, it also has two shortcomings:

(1) Fewer constraints are available in the phase of annotating the last hops.

When the last hop of a traceroute path is not the destination, it may be because the destination AS is configured with a firewall to prevent it from responding to other networks^11^. In this case, bdrmapIT considers that the last interface may be on the border router of the network containing the destination. So in the stage of annotating the last hops, bdrmapIT infers the operator of a router with no outgoing links to be its destination AS of the paths on which the router’s interfaces are observed. Since only the destination AS set is used as the candidate set, relatively few constraints are available. bdrmapIT may have some misjudgments for such routers. To better handle this type of router, it’s feasible to enrich topological constraints for inferring routers’ owners.

(2) The failure of experience can lead to errors.

The bdrmapIT relies on empirical knowledge in Internet practice when mapping routers to their operating AS and infers the results by designing various rules. In practical applications, this rule-based approach can handle problems in some scenarios. Still, some exceptions are not defined by the rules, which will affect the processing effect of the method. For example, bdrmapIT may choose candidate ASes based on the valley-free assumption^19^, but there are violations of the valley-free rule.

### Problem definition

To improve the shortcomings of bdrmapIT, we consider adding available constraints for router ownership inference, that is, different types of IP links (inter-domain links and intra-domain links). Here, we first introduce related definitions and concepts. Then, our problem is formally defined.

#### Definition 1

*(Path Information):* $p=IP_{1}, \ldots , IP_{n}$, where $n \ge 2$ represents an IP path. $P=\left\{ p_{1}, p_{2}, \ldots , p_{n}\right\}$ represents a collection of all paths included in the traceroute data.

#### Definition 2

*(IP Link):* Each path can be divided into several end-to-end IP links $\left\langle IP_{i}, {IP_{i+1}} \right\rangle$, where $1 \le i \le n-1$.

#### Definition 3

*(Network Topology):* IP-level Topology ${G_{IP}}$: ${G_{IP}} = \left( {V_{IP},{E_{IP}}} \right)$, where $V_{IP}$ represents the set of IP nodes including all IP addresses on paths in *P*, and $E_{IP}$ represents the set of edges composed of IP node pairs. $G_{IP}$ is constructed by merging all paths in *P*.

Router-level Topology ${G_R}$: ${G_R} = \left( {{V_R},{E_R}} \right)$, where $V_R$ represents the set of router nodes, and $E_R$ represents the set of edges composed of router node pairs. $G_R$ can be generated from $G_{IP}$ through alias resolution.

#### Definition 4

*(Mapping Function):* IP-to-Router mapping ${I_R}:V_{IP} \rightarrow {V_R}$ represents the mapping from IP addresses to logical router identifiers.

IP-to-AS mapping ${I_{AS}}:V_{IP} \rightarrow {S_{AS}}$ represents the mapping from IP addresses to ASNs, ${S_{AS}}$ means the set of ASNs.

Router-to-AS mapping ${R_{AS}}:{V_R} \rightarrow {S_{AS}}$ represents the mapping from logical router identifiers to ASNs.

#### Definition 5

*(Intra-domain Link):* The intra-domain link refers to the link between interfaces on routers in the same AS, that is,

$$\begin{aligned} \left\langle I{{P}_{i}},I{{P}_{i+1}} \right\rangle ,{{R}_{AS}}\left( {{I}_{R}}\left( I{{P}_{i}} \right) \right) ={{R}_{AS}}\left( {{I}_{R}}\left( I{{P}_{i+1}} \right) \right) . \end{aligned}$$

#### Definition 6

*(Inter-domain Link):* The inter-domain link refers to the link between interfaces on two border routers of connected ASes, that is,

$$\begin{aligned} \left\langle I{{P}_{i}},I{{P}_{i+1}} \right\rangle ,{{R}_{AS}}\left( {{I}_{R}}\left( I{{P}_{i}} \right) \right) \ne {{R}_{AS}}\left( {{I}_{R}}\left( I{{P}_{i+1}} \right) \right) . \end{aligned}$$

The problem studied in this paper is: given a traceroutes set *P*, generate an IP-level topology ${G_{IP}}$ and a router-level topology ${G_{R}}$; combining the IP-to-AS mapping ${I_{AS}}$, design features to classify IP links; on this basis, use the additional information of the link type to enrich inference constraints, and accurately map ***last-AS*** routers to their owner ASes. The key stages involved include the design of IP link features, the classification of intra-domain and inter-domain links, and the inference of router ownership.

## Proposed LCROI method

IP links can be classified into two types: inter-domain links and intra-domain links. Each kind of link has some observable features, and the probabilities of particular features occurring are different for various link types. The type of IP links in the IP-level topology derived by the traceroute probe is unknown. Still, appropriate features can be designed based on domain knowledge to distinguish between inter-domain and intra-domain links. Due to the anonymous nature of IP link types and the different probabilities exhibited by features, the IP link classification problem can be modelled as a probabilistic model with IP link type as the hidden variable, and the probabilities of given features occur for different link types as parameters.

The classification of IP link types relies on the accurate prediction of probabilistic model parameters. The Expectation-Maximization (EM) algorithm is suitable for estimating probability models with hidden variables. Therefore, in our problem, due to the existence of the hidden variable (IP link types), the EM algorithm is used to estimate the parameters iteratively. In each iteration of the EM algorithm, considering the possible correlation among features, we use the Naive Bayes classification technique^20^. The EM algorithm can converge and give a set of stable parameters for the Naive Bayes classifier. Therefore, it is feasible to use Naive Bayes as the classification technique.

**Figure 2:**
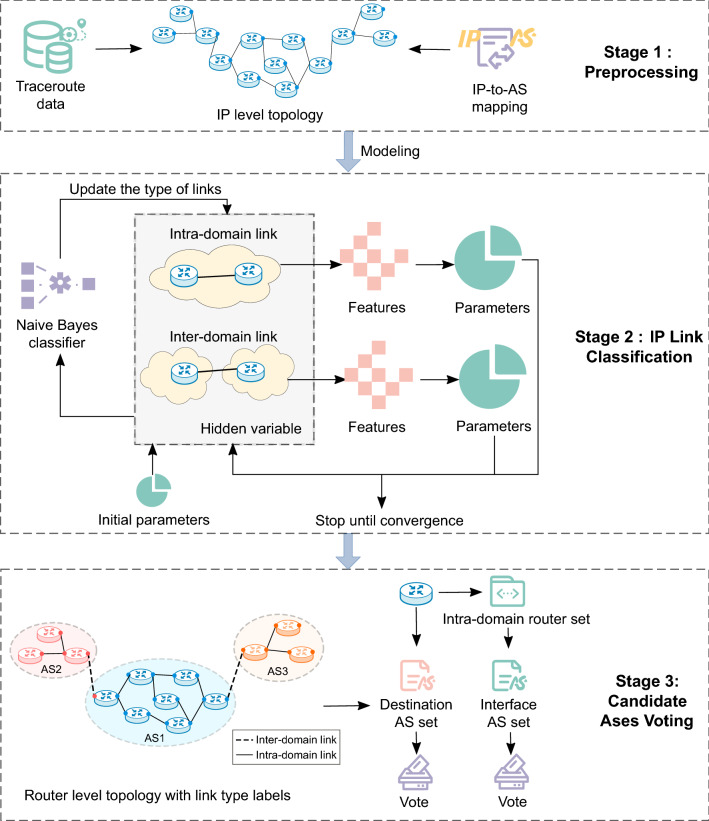
The process of the proposed LCROI method.

In summary, this paper proposes a router ownership inference method based on the classification of intra- and inter-domain links. The main idea is to design distinguishing features, use a probability model to classify IP links, and determine owner ASes of last-AS routers based on the voting mechanism. The principle framework of the method is shown in Fig. 2. It mainly includes three stages: preprocessing, IP link classification, and candidate ASes voting. The preprocessing stage includes path preprocessing, network topology construction, and IP-to-AS mapping. The IP link classification stage is based on the probabilistic model and uses carefully designed features to divide all IP links into two types: inter-domain links and intra-domain links. The candidate ASes voting stage uses two candidate AS lists to vote together to infer routers’ operating ASes.

The main steps of the method are as follows:

- *Step 1: Path preprocessing.* First, to avoid mistaking the host as a router, the destination addresses of traceroute paths are ignored. That is, when extracting IP addresses from traceroute paths for alias resolution, only addresses that are intermediate hops in some paths are covered. After that, delete paths with only one IP address and paths with IP loops. In addition, delete IP addresses belonging to the BGP route server ASN provided by IXP for establishing multilateral peer-to-peer connections.
- *Step 2: Network topology generation.* Use the path processed in step 1 to generate an IP-level network topology.
- *Step 3: IP-to-AS mapping.* Use IP-to-AS mapping technology to map all IPs in the IP-level topology to their origin ASNs. For MOAS, to choose “the best” mapping for simplicity, the AS that appears as an origin AS most frequently in the source BGP table can be selected^21^.
- *Step 4: Modeling.* Model the IP link classification problem as a probabilistic model with IP link type as the hidden variable, and the parameters are the probabilities of particular features occurring for different link types.
- *Step 5: IP link classification.* Use the EM algorithm to estimate the parameters of the model, and the Naive Bayes classifier is used to calculate the probability that the IP link belongs to each category. After the convergence is reached, output the result according to the probability and threshold.
- *Step 6: Candidate ASes voting.* According to the IP link classification result obtained in step 5, the corresponding router-level link type is determined. A router-level topology containing link-type labels is constructed after alias resolution. Generate an intra-domain router set for each router, and combine the destination AS set to vote together to determine its owner AS.

In the above steps, the key steps are IP link feature design in modelling, IP link classification, and candidate ASes voting, which are described in detail below.

### IP link feature design

Based on domain knowledge, this section designs three features that distinguish between inter-domain and intra-domain links, including the AS relation feature of adjacent IP links, the fan-in and fan-out features, and the IP address vector distance feature of IP links.

#### AS relation feature of adjacent IP links

The link type also relates to the business relationship between ASes. Without considering the complex hybrid relationship, AS relationships can be categorized as provider-to-customer (p2c), customer-to-provider (c2p), peer-to-peer (p2p), and sibling-to-sibling (s2s)^22^.

In our method, we use ${{I}_{AS}}\left( IP_{i} \right)$ to denote the AS that $IP_{i}$ is mapped to. According to the different types of IP addresses and whether there is an IP-to-AS mapping result, the value of ${{I}_{AS}}\left( I{{P}_{i}} \right)$ has 4 possible situations in this paper, as shown in Table 2.

**Table 2:** Values of ${{I}_{AS}}\left( IP_{i} \right)$.

$IP_{i}$	${{I}_{AS}}\left( IP_{i} \right)$
IP addresses used in Local Area Network	−1
IP addresses belong to IXP prefixes	Less than −1 and associated with the IXP ID in PeeringDB
Other IP addresses with IP-to-AS mapping results	Corresponding AS number (positive value)
IP address without IP-to-AS mapping result	Missing

$REL\left( <IP_{i}, IP_{i+1}>\right)$ is utilized to denote the AS relationship between $I_{AS}\left( IP_{i}\right)$ and $I_{AS}\left( IP_{i+1}\right)$. Besides the three relations above, we annotate the IP link $<IP_{i}, IP_{i+1}>$ with the label ‘*same*’ if $I_{AS}\left( IP_{i}\right) =I_{AS}\left( IP_{i+1}\right)$. In addition, AS relationships involving IP addresses used in Local Area Networks (LAN), IXP prefixes, and IP addresses without origin AS result are also indicated by corresponding labels, as shown in Table 3.

**Table 3:** Values of $REL\left( <IP_{i}, IP_{i+1}>\right)$.

${{I}_{AS}}(IP_{i})$	${{I}_{AS}}(IP_{i+1})$	relationship between ${{I}_{AS}}(IP_{i})$ and ${{I}_{AS}}(IP_{i+1})$	$REL\left( <IP_{i}, IP_{i+1}>\right)$
Missing	Missing	N/A	(*missing*, *missing*)
Mssing	−1	N/A	$(missing, -1)$
Missing	$<-1$	N/A	$(missing, <-1)$
Missing	Positive value	N/A	(*missing*, *positive*)
−1	-1	N/A	$(-1, -1)$
−1	$<-1$	N/A	$(-1, <-1)$
−1	Positive value	N/A	$(-1, positive)$
$<-1$	$<-1$	N/A	$(<-1, <-1)$
$<-1$	Positive value	N/A	$(<-1, positive)$
Positive value	Positive value	$I_{AS}\left( IP_{i}\right) =I_{AS}\left( IP_{i+1}\right)$	Same
Positive value	Positive value	p2c	p2c
Positive value	Positive value	c2p	c2p
Positive value	Positive value	p2p	p2p
Positive value	Positive value	s2s	s2s

With regard to the IP link $<IP_{i}, IP_{i+1}>$, its ***forward AS relation feature*** is the set

$$\begin{aligned} \left\{ \left( REL\left(<IP_{i-1}, IP_{i}>\right) , REL\left( <IP_{i}, IP_{i+1}>\right) \right) \right\} , \end{aligned}$$

where the tuple $\left( REL\left(<IP_{i-1}, IP_{i}>\right) , REL\left( <IP_{i}, IP_{i+1}>\right) \right)$ denotes the relation of AS relationship between IP link $<IP_{i}, IP_{i+1}>$ and its forward link $<IP_{i-1}, IP_{i}>$ in path *p*. The *forward AS relation* set is obtained after traversing all paths *P*. Similarly, the ***backward AS relation feature*** of IP link $<IP_{i}, IP_{i+1}>$

$$\begin{aligned} \left\{ \left( REL\left(<IP_{i}, IP_{i+1}>\right) , REL\left( <IP_{i+1}, IP_{i+2}>\right) \right) \right\} , \end{aligned}$$

can also be yielded.

**Figure 3:**
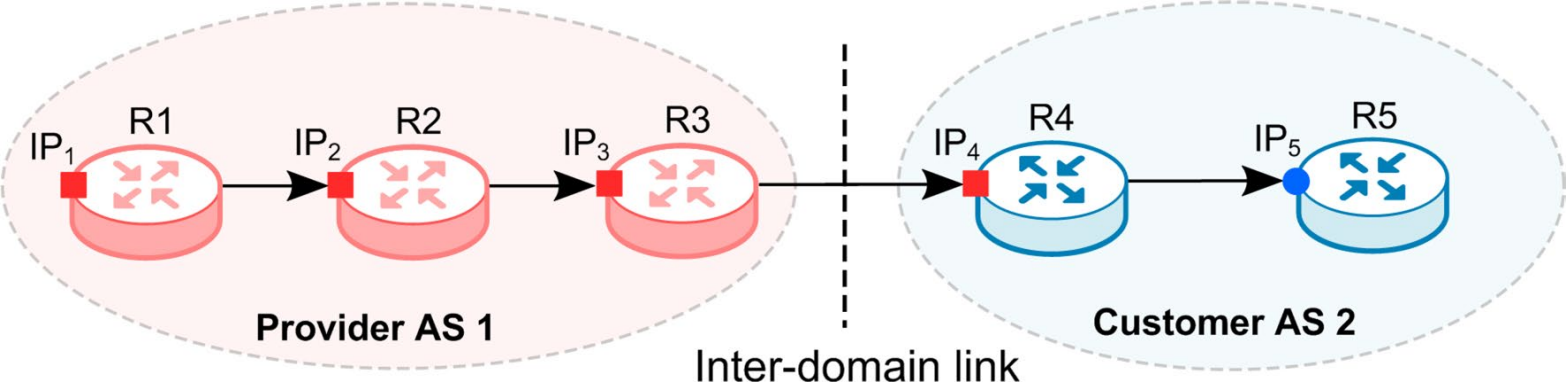
Example of p2c link in which the provider supplies the address space.

We take a traceroute path as an example to analyze the role of the AS relation feature of adjacent IP links in the classification of IP links. As shown in Fig. 3, a traceroute path $IP_{1}(R1,AS1) \rightarrow IP_{2}(R2,AS1) \rightarrow IP_{3}(R3,AS1) \rightarrow IP_{4}(R4,AS1) \rightarrow IP_{5}(R5,AS2)$, where $IP_{i}(Ri,ASj)$ denotes that $IP_{i}$ on the router *Ri* was mapped to *ASj*. In Internet practices, when two ASes connect with each other by the point-to-point link, the IP subnet of the link (usually a /30 or /31 in IPv4) is typically from one of the two ASes’ address space^23^. It is worth noting that in p2c relationships, the provider usually supplies the address space for the IP subset. Moreover, when passing a p2c link in a traceroute probe, the customer’s router usually uses the IP address from the provider’s space to respond to the probe. In the example, AS1 is the provider of AS2, and AS1 provides the address space for the IP link between them, i.e., the inter-domain link in fact is $<IP_{3}, IP_{4}>$, rather than $<IP_{4}, IP_{5}>$ where the ASNs seems to change. As a result, for an IP link $<IP_{i}, IP_{i+1}>$, if $({\textit{same}, } p 2 c)$ is included in its *forward AS relation feature*, it may be an inter-domain link. Furthermore, if (*same*, *same*) both in the *forward* and *backward* *AS relation feature* of a link, the link is likely to be an intra-domain link.

#### Fan-in and fan-out features

**Figure 4:**
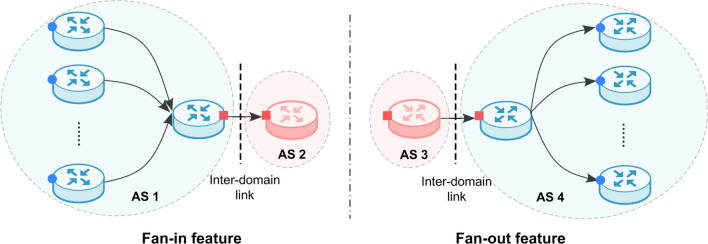
Illustrations of fan feature.

In general, multiple probe paths passing through different routers in an AS will converge to the border router and cross the inter-domain link to enter its neighbor AS. Similarly, multiple probe packets traversing the inter-domain link to reach an AS will be distributed and forwarded to different routers inside the AS. Therefore, for an inter-domain link, it may have more previous or subsequent links with AS switch (as shown in Fig. 4) than an intra-domain link. The fan feature captures the likelihood of a certain number of links where an AS switch occurs before (***fan-in feature***) or after (***fan-out feature***) a particular link given its link type.

For each IP link $<IP_{i}, IP_{i+1}>$, traverse all traceroute paths and count the number of forward links $<IP_{i-1}, IP_{i}>$ (backward links $<IP_{i+1}, IP_{i+2}>$) with an AS switch in paths including $<IP_{i}, IP_{i+1}>$ as the *fan-in (fan-out) feature* of the link.

#### IP address vector distance feature

**Figure 5:**
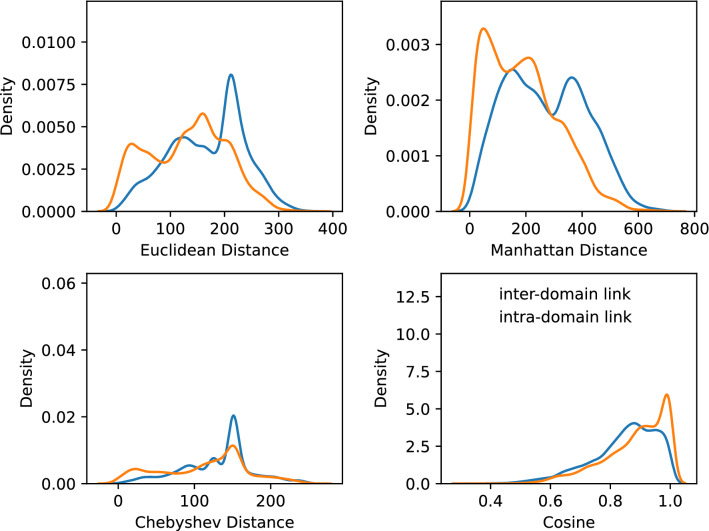
The vector distance statistic histogram of IP addresses at both ends of IP links belong to two different types.

Considering that IP addresses within the same AS may be in the same or similar IP prefixes, while IP addresses belonging to different ASes may be far apart, the IP address vector distance feature is proposed. Specifically, the IP(v4) address is a 32-bit binary number that is typically split into four bytes. For each IP link $<IP_{i}, IP_{i+1}>$, expressing $IP_{i}$ and $IP_{i+1}$ as vectors yields $I P_{i}=\left[ x_{1}, x_{2}, x_{3}, x_{4}\right]$ and $I P_{i+1}=\left[ y_{1}, y_{2}, y_{3}, y_{4} \right]$, where $x_{j}$ and $y_{j}$ denote the *j*th byte of $IP_{i}$ and $IP_{i+1}$, respectively. Count the Euclidean distance, Manhattan distance, Chebyshev distance, and cosine of some IP vectors at both ends of intra-domain links and inter-domain links obtained by using the bdrmapIT method^11^. The histogram is shown in Fig. 5.

It can be seen from Fig. 5 that the Euclidean distance (or Manhattan distance) between the IP addresses at both ends of the inter-domain link is generally greater than that of the intra-domain link. Therefore, the ***IP address vector distance feature*** is used to indicate that the two IPs at the ends of an inter-domain link typically have a larger Euclidean distance than that of an intra-domain link. The *IP address vector distance feature* of the IP link $<IP_{i}, IP_{i+1}>$ is calculated as the following Eq. (1).

1 $$\begin{aligned} Euclidean\left( IP_{i}, IP_{i+1}\right) =\sqrt{\sum _{j=1}^{4}\left( x_{j}-y_{j}\right) ^{2}} \end{aligned}$$

### IP link classification

We use the EM algorithm to estimate the parameters of the probability model established in this paper. Specifically, first, calculate the conditional probability distribution of each feature for different link types, then use the Naive Bayes classifier to update the type of each link, and recalculate the feature distribution according to the new probability. Repeat the above two steps until convergence.

The probabilistic model requires initial parameters. Therefore, first, simply use the IP-to-AS mapping data to generate initial type labels (intra-domain links or inter-domain links) for all IP links. The specific method is to consider the link without AS switch as an intra-domain link, and the link with AS switch as an inter-domain link. In addition, it is believed that the provider provides the address space used by the interconnection in the p2c link.

The following pseudocode introduces the implementation of the probabilistic IP link classification algorithm.
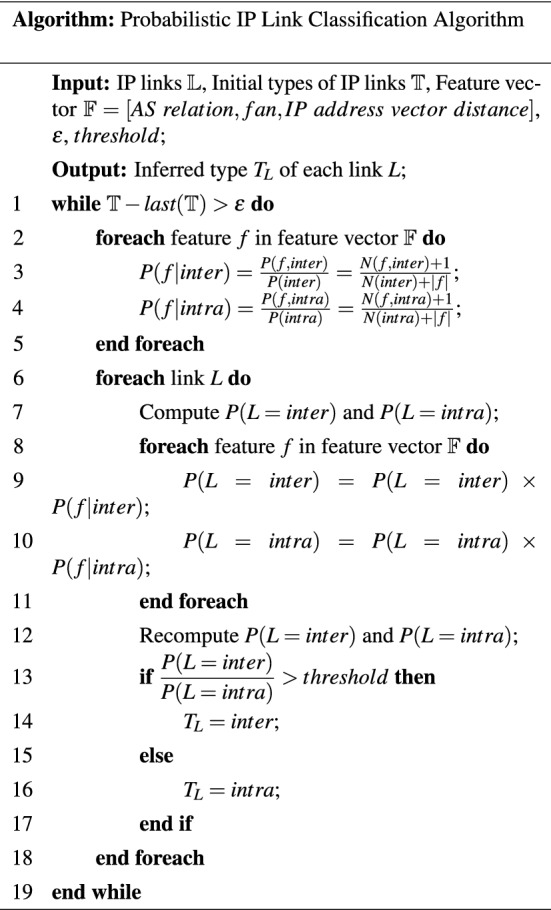


The input of the algorithm is all IP links, initial types of IP links, the feature vector, a minimum value $\varepsilon$ and the *threshold*. The output is the inferred type of each IP link. Firstly, for each feature in the feature vector, we calculate the conditional probability of the two link types separately. To solve the problem of zero probability, the algorithm uses Laplace (Add-1) Smoothing^24^ (Lines 2-5). We then compute the prior probability of the links’ types by counting the proportion of the two link types at the beginning. The prior probability of each link type is then multiplied by the conditional probability of all the features (Lines 6-11). Finally, recalculate the probabilities of the two link types. When the ratio of the probability of the inter-domain link to the intra-domain connection is greater than the threshold, it is inferred that the link is an inter-domain link; otherwise, it is an intra-domain link (lines 12-17). The process of link type inference and updating conditional probability distributions of features are repeated until convergence, i.e., the number of links that change the type label between the current iteration and the prior iteration decrease to a small value $\varepsilon$. The time complexity of the algorithm is *O*(*e*), where *e* is the number of IP links in the network topology.

### Candidate ASes voting

A router may have many interfaces, and a router-level link may have many corresponding IP links. For a router-level link, if one of its corresponding IP links is an inter-domain link, we think that the router-level link is an inter-domain link; otherwise, it is an intra-domain link. After assigning all the router-level links a type label, all the routers are divided into different ASes by inter-domain links in the router-level topology.

For a router, we call neighbor routers connected to it with the intra-domain link *intra-domain neighbors*, while neighbor routers connected to it with the inter-domain link are called *inter-domain neighbors*. For a given router *R*, we first find its *intra-domain neighbors*. For the *intra-domain neighbors*, we choose routers without *inter-domain neighbors*, and continue to find their *intra-domain neighbors*. We repeat the process until there are no routers eligible for the requirement mentioned above. All the *intra-domain neighbors* found are added to the *intra-domain router set* $S_{R}$ of the given router *R*.

Let $S_{IP}(R)$ denote the set of all IP interfaces on *R*. We generate a list of candidate ASes $CL_{N}(R)$ for *R*, containing the ASes to which IPs of all routers in $S_{R}$ belong, i.e.,

2 $$\begin{aligned} CL_{N}(R)=\left\{ I_{AS}(IP_{i})|IP_{i} \in S_{IP}(R),R \in S_{R} \right\} \end{aligned}$$

where the ASNs can be repeated.

The set of traceroute destination addresses of all paths containing $IP_{i}$ is denoted as $DS_{I}(IP_{i})$. The union of destination address sets of all IPs on router *R* is represented by $DS_{R}(R)=\left\{ DS_{I}(IP_{1}) \cap ... \cap DS_{I}(IP_{n}) \right\}$, where $IP_{i} \in S_{IP}(R)$, $1 \le i \le n$.

We can generate another candidate ASes list $CL_{D}(R)$ for *R* consisting of ASes that all IPs in $DS_{R}(R)$ are mapped to, i.e.,

3 $$\begin{aligned} CL_{D}(R)=\left\{ I_{AS}(IP_{i})|IP_{i} \in DS_{R}(R) \right\} \end{aligned}$$

where the ASNs are repeatable.

We use the voting method to determine the AS annotation for *R*. The election is held among ASNs in the list $CL_{N}(R)$ and $CL_{D}(R)$ separately. When an AS is the winner of both $CL_{N}(R)$ and $CL_{D}(R)$, we assign *R* to this AS.

## Experiments and results

To verify the performance of the proposed method, we conducted experiments using publicly available topology probe data.

### Experimental settings

This section introduces the experimental environments and tools, the public datasets used in the experiments, the methods for comparison, and the evaluation metrics of the experimental performance.

#### Experimental environments and tools

The experiment results were obtained using a personal computer with an Intel Core i5-7400 Processor (3.5 Gigahertz), 4 Cores, and 16 Gigabytes of memory, running Windows 10 64-bit operating system. Additionally, when using Team Cymru’s IP to ASN mapping service, we used an AliCloud server running the CentOS 7 operating system with 2 virtual central processing units and 4 Gigabytes of memory. The programming language we utilized is python, and the integrated development environment is JetBrains PyCharm community edition 2019.1.2.

#### Experimental datasets

The experiment requires traceroute probe data, IP-to-AS mapping data, IXP prefix list, routing server ASN list, AS relationship data, and verification set data used to verify the inference results.

The primary experimental data mainly come from some Internet research institutions, management institutions, projects, and databases, including the Center for Applied Internet Data Analysis (CAIDA)^25^, RouteViews^26^, Team Cymru^27^, Regional Internet Registry (RIR)^28^, Euro-IX^29^, PeeringDB^30^, etc.

To verify the AS ownership inference result is not easy. The reason is that operators generally hide their network topology to avoid possible network security risks and commercial competition risks. Therefore, in the public data, the data in PeeringDB is first selected as the verification set. PeeringDB is an open, user-maintained network interconnection database designed to support the actual needs of network operators, but it is also a valuable source of information for researchers. In PeeringDB, we can find out the real member ASN of the router whose IP interface address is mapped to the IXP operator. In addition, researchers found that by analyzing the Domain Name System (DNS) host name, some interface information of the router can be obtained, which helps to determine the type and location of the router^31^. Therefore, we also manually generate a verification set based on the DNS hostname data, that is, the TeliaSonera (AS1299)^32^ verification set. The reason for choosing this AS is that its DNS host names used for inter-AS connections often indicate the name of the interconnected ASes^33^.

Specifically, the TeliaSonera verification set is manually generated by selecting interfaces in the paths associated with TeliaSonera. An example is interface *62.115.14.14* with the hostname *planetel-ic-301155-mno-b2.c.telia.net*. It’s mapped to AS1299 in IP-to-AS mapping data, but in fact, its router belongs to AS47217 whose AS name is *PLANETEL,IT*. So we find hostnames containing the string ‘*-ic*’, and match the string before ‘*-ic*’ with the names of all the ASes that may connect to TeliaSonera. On the other hand, for hostnames without the above-mentioned character (like *snn-sec1-link.se.telia.net*), we think these interfaces, as well as their routers, are in TeliaSonera. We can’t decode every hostname since some hostnames have no hints or exist ambiguous information.

Finally, to verify the IP link classification results, a verification set (TeliaSonera-1) containing a total of 26755 IP links is generated, of which 16,180 are inter-domain links and 10,575 are intra-domain links. To verify the AS ownership inference result, a verification set (TeliaSonera-2) containing a total of 2727 routers is generated, of which 1157 are in TeliaSonera and 1570 are in other ASes connected to it.

The data sources, data scales, and main applications in the experiment are shown in Table 4.

**Table 4:** The data sources, data scales, and main applications in the experiment.

Data	Data source	Data scale	Main application
Traceroute	CAIDA Ark Team1 (cycle 7131 to 7159)^34^	295,408,669 paths	Basic data; build network topology
ITDK	Macroscopic Internet Topology Data Kit in CAIDA^35^	Two related IPv4 router-level topologies; router-to-AS assignments; DNS lookups of all observed IP addresses	Alias resolution; experiment result comparison
IP-to-AS mapping	Prefix-to-AS mapping data from RouteViews^36^; Team Cymru IP-to-AS mapping tool^37^; Regional Internet Registry^28^	3,074,320 mappings	Pretreatment
IXP prefixes	IXP data in CAIDA^38^	853 prefixes	Pretreatment
Route server ASNs	PeeringDB; Euro-IX	184 ASNs	Pretreatment
AS relationship	Inferred by ProbLink^22^; CAIDA^39^	688078 pairs	Design IP link features
PeeringDB validation set	PeeringDB^30^	16864 routers	Verify experiment results
TeliaSonera-1 validation set	TeliaSonera (AS1299)^32^	26755 IP links	Verify experiment results
TeliaSonera-2 validation set	TeliaSonera (AS1299)^32^	2727 routers	Verify experiment results

#### Methods for comparison

Here, we introduce two baseline methods in detail: MAP-IT method^33^, and bdrmapIT method^11^. As far as we know, the MAP-IT method is a classic method to infer inter-domain links between ASes. It is verified with real data, and the result has a high accuracy rate. The bdrmapIT method is a new method that implements router ownership inference on the Internet scale. When it mapped AS boundaries on two traceroute data sets at the Internet scale, the accuracy is more than 90%.

**MAP-IT** MAP-IT tries to infer the exact interface addresses used for point-to-point inter-AS links, as well as the specific ASes involved. MAP-IT combines evidence of AS switches from distinct traceroute traces, using the forward and backward neighbors set of each interface to infer. Each pass leverages prior inferences to refine existing inferences and to discover additional inter-AS link interfaces. Although MAP-IT does not include the alias resolution process, its inference is the AS of the interface’s router. Since MAP-IT only deduces the inter-AS links, for internal routers of ASes, we determined their ASes by IP-to-AS mapping.

**bdrmapIT** The detailed introduction of the bdrmapIT method is as described in the “Analysis of bdrmapIT” section above.

#### Evaluation indicator

For a router, the correct inference is mapping it to its operating AS. Conversely, if a router is assigned to an AS different from its real owner AS, the inference is considered to be incorrect. The evaluation metric in this experiment is the *accuracy*, i.e., the fraction of routers with correct inferences:

4 $$\begin{aligned} accuracy=\frac{correct \ \ inferences}{all \ \ inferences} \times 100\% \end{aligned}$$

### Result analysis of IP link classification

The accuracy of the IP link classification result would affect the AS ownership inference in the subsequent steps, so this subsection verifies it experimentally. The effect of IP link classification was verified on the TeliaSonera-1 validation set.

The last step in the algorithm determines the result according to the relation between probabilities and the threshold, that is, if $P(L=inter) / P(L=intra) > threshold$, a link is predicted to be an inter-domain link. The inferred results can be classified into four categories as follows:

- True Positive (TP): both the inferred type and the true type are inter-domain links.
- False Positive (FP): the inferred type is the inter-domain link, while the true type is the intra-domain link.
- True Negative (TN): both the inferred type and the true type are intra-domain links.
- False Negative (FN): the inferred type is the intra-domain link, while the true type is the inter-domain link.

**Figure 6:**
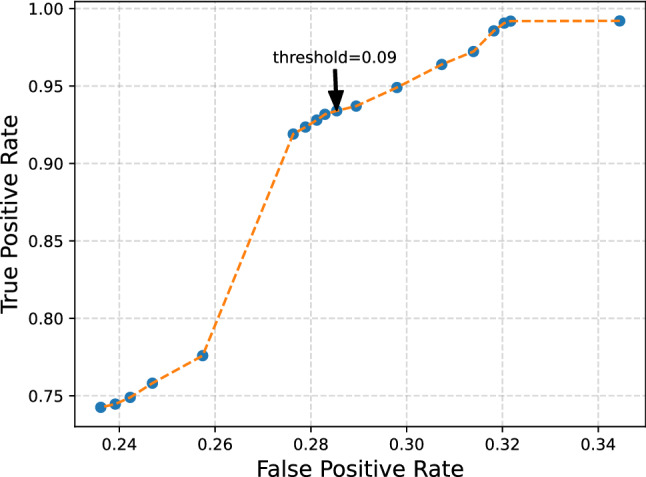
ROC curve diagram of IP link classification under different thresholds.

The effect of the algorithm is related to the selection of the threshold in the last step. Therefore, we used the receiver operating characteristic curve (ROC curve) to improve the decision rule by observing the different effects of the algorithm for different thresholds. The local ROC curve is shown in Fig. 6. The x-axis represents the false positive rate, and the y-axis represents the true positive rate. It can be seen from the figure that the algorithm has a high true positive rate and a low false positive rate for the classification results of intra-domain and inter-domain links, and the overall classification effect is good. Besides, when the threshold is less than 0.09, the overall true-positive rate increases rapidly and the false-positive rate increases slowly. When the threshold is greater than 0.09, the false positive rate increases rapidly as the true positive rate increases slowly. To obtain both a high true positive rate and a low false positive rate, the final threshold selected in the experiment is 0.09.

With a determined threshold of 0.09, *Precision*, *Recall*, and *F1 Score* are selected as metrics to assess the performance, reflecting its ability to classify IP links. After counting the numbers of the above four cases respectively, *Precision* can be interpreted as how many links predicted to be inter-domain links are true inter-domain links, as shown in Eq. (5).

5 $$\begin{aligned} Precision =\frac{TP}{TP+FP} \end{aligned}$$

*Recall* means how many of true inter-domain links are correctly inferred, as shown in Eq. (6).

6 $$\begin{aligned} Recall =\frac{TP}{TP+FN} \end{aligned}$$

*F1 Score* is the weighted average of Precision and Recall, which is shown in Eq. (7).

7 $$\begin{aligned} F1 \ Score =\frac{2 \times Precision \times Recall}{Precision+Recall} \end{aligned}$$

The numbers of IP links about different circumstances and the classification effect of the algorithm are shown in Table 5. Experimental results on the dataset of 26755 IP links demonstrate that the IP link classification algorithm achieves a high precision and recall rate. During subsequent router ownership inference steps, the types of links between routers can provide more topological constraints to help accurately infer.

**Table 5:** The result of IP link classification algorithm on the verification set TeliaSonera-1.

TP	FP	TN	FN	Precision	Recall	F1 Score
15112	3018	7557	1068	83.4%	93.4%	88.1%

### Stability analysis of candidate ASes voting

In the candidate AS voting stage, there may be two scenarios in the voting process: a tie or a non-tie. In the case of a non-tie, a unique voting winner is selected, but in the tie scenario, more than one AS will win. The handling of the tie situation may affect the final result of the router ownership inference.

In the experiment, when the vote was tied, we chose randomly among ASes with the most votes. Therefore, we ran our program ten times to consider the effect of the random selection strategy on the accuracy of router ownership inference results. The validation sets for the experiments were PeeringDB and TeliaSonera-2. The results are shown in Table 6.

**Table 6:** The accuracy of router ownership inference results using random selection strategy.

No.	# all inferences		# correct inferences		accuracy	
	PeeringDB^30^	TeliaSonera-2^32^	PeeringDB^30^	TeliaSonera-2^32^	PeeringDB^30^	TeliaSonera-2^32^
1	6842	552	6588	522	96.3%	94.6%
2	6824	544	6580	516	96.4%	94.9%
3	6803	545	6560	517	96.4%	94.9%
4	6811	549	6565	517	96.4%	94.2%
5	6824	554	6576	523	96.4%	94.4%
6	6788	548	6545	517	96.4%	94.3%
7	6798	547	6553	519	96.4%	94.9%
8	6810	549	6563	519	96.4%	94.5%
9	6843	543	6595	516	96.4%	95.0%
10	6819	550	6572	519	96.4%	94.4%

It shows that the accuracy of the results is stable at around 96.4% and 97.8% on the two validation sets, respectively. This experiment indicates that the random selection strategy has a slight effect on the number of routers with inferences, but almost no effect on their accuracy. That is, our strategy for the candidate ASes has high accuracy and stability.

### Comparison with other methods

**Figure 7:**
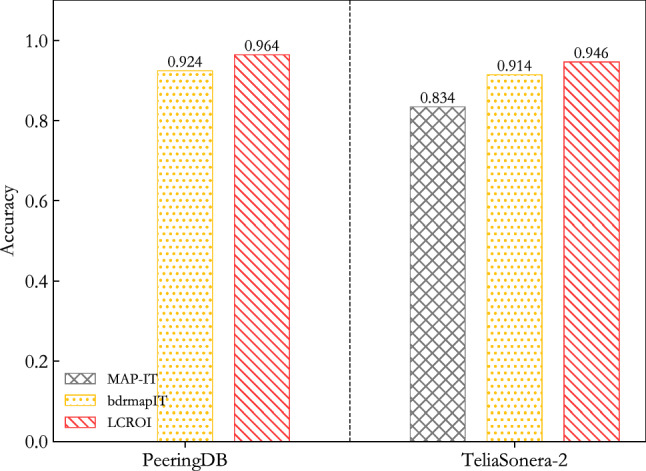
Comparison between LCROI and other methods using MIDAR^40^ and iffinder^41^ for alias resolution.

This section compares LCROI with MAP-IT and bdrmapIT on the accuracy indicator, and the experimental results are shown in Fig. 7. The data in the figure are the average results of 10 experiments.

It can be seen from Fig. 7 that the average accuracy of LCROI is 96.4% on PeeringDB validation set, which is better than bdrmapIT’s 92.4%, increasing 4%. Since the MAP-IT method does not infer the owner AS of IP addresses in IXP prefixes, it cannot be compared with it on the PeeringDB verification set. The average accuracy of LCROI is 94.6% on TeliaSonera-2 validation set, which exceeds 83.4% of MAP-IT, and 91.4% of bdrmapIT with the growth of 11.2% and 3.2%.

We also studied the impact of different alias resolution methods on the accuracy of our approach. The ITDK dataset contains two router-level topologies generated from the same IP-level topology using different alias resolution techniques. The first topology uses MIDAR^40^ and iffinder^41^ for alias resolution, and the second topology uses kapar^42^ besides MIDAR and iffinder. The two topologies differ in accuracy and completeness. The former has high confidence and resolves a few false positives in aliases, while the latter enriches the aliases but with more false positives.

**Figure 8:**
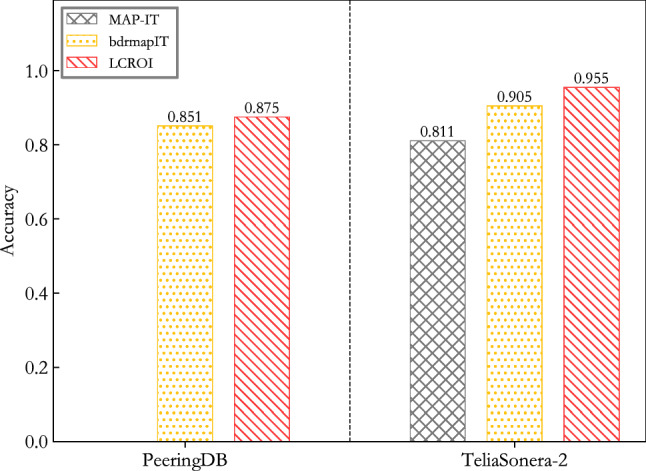
Comparison between LCROI and other methods using MIDAR^40^, iffinder^41^, and kapar^42^ for alias resolution.

The results in the above experiment use only midar and iffinder techniques for alias resolution. To determine the effect of less accurate but more enriched aliases on the experimental results, we used the second topology in ITDK to experiment. The results are shown in Fig. 8.

It can be seen from Fig. 8 that the average accuracy of LCROI is 87.5% on PeeringDB validation set, which is better than bdrmapIT’s 85.1%, increasing 2.4%. The average accuracy of LCROI is 95.5% on TeliaSonera-2 validation set, which exceeds 81.1% of MAP-IT and 90.5% of bdrmapIT with the growth of 14.4% and 5%.

In general, the inference effect of LCROI is better than the two comparison methods. In particular, we analyze the possible reasons why LCROI can correct the wrong result of bdrmapIT.

For routers wrongly inferred in *annotating last hops* stage, since they appear only at the last hop of paths, bdrmapIT has no topological constraints other than the set of destination ASes for router ownership inference in its second stage, leading to some misjudgments of such routers. LCROI uses the router’s *intra-domain router set* and traceroute destination addresses to jointly construct lists of candidate ASes for voting, adding topological constraints to infer the routers mentioned above more accurately.

There could be the following reasons responsible for incorrectly inferred routers in the stage *graph refinement*. First, the type of links cannot be taken into account in bdrmapIT when summing the subsequent interfaces of routers, resulting in inter-domain neighbors being a possible interfering factor. If the number of inter-domain neighbors is higher than the number of intra-domain neighbors, a wrong inference may generate. Our method classifies the IP links and reduces confounding factors when voting. Second, the failure of experience can lead to errors. For example, when a router’s subsequent interface in a traceroute path is an IXP public peer address, bdrmapIT typically selects the candidate AS with the largest customer-cone based on traditional assumptions, ignoring violations of *valley-free* property, thus leading to the existence of misjudgments. Our approach reveals the characteristics of the data themselves rather than only relying on subjective judgments from experience, improving such distortions to some extent.

## Conclusion and future work

We designed the IP address vector distance feature, the AS relationship feature of the IP link, and the fan-in and fan-out features to distinguish intra-domain links and inter-domain links. Based on this, we presented a probability model with the IP link type as the hidden variable to classify IP links. The use of the additional information derived from the link type enriches the basis for router ownership inference, and improves the accuracy of the inference result. Using two separate validation datasets to verify the result, the accuracy of our method ranges between 94.6% and 96.4%, which is slightly better than MAP-IT and bdrmapIT. We think that the more representative features may bring better classification performance on types of IP links, and further optimize the router ownership inference.

In future work, we will pay attention to features that provide better classification effects of IP links. The complex hybrid AS relationships are also worth exploring. In addition, future research should be conducted in more realistic settings to further develop and confirm these initial findings.

## Data Availability

The datasets generated and analysed during the current study are available in the CAIDA, RouteViews and Team Cymru repository: The CAIDA ucsd ipv4 routed /24 topology dataset - dec 26, 2018 to jan 10, 2019. http://www.caida.org/data/active/ipv4_routed_24_topology_dataset.xml. The CAIDA ucsd internet topology data kit (itdk) - jan 1, 2019. https://www.caida.org/data/internet-topology-data-kit. IP-to-ASN mapping data. http://www.routeviews.org/. http://www.team-cymru.org/IP-ASN-mapping.html. The CAIDA ucsd IXPs dataset, jan, 2019. https://www.caida.org/data/ixps. The CAIDA AS relationships dataset - jan 1, 2019. http://www.caida.org/data/active/as-relationships/.

## Code availability

The code for data processing and experiments associated with the current submission is available at https://github.com/fuliOp/LCROI.git.
